# The characterization, management, and future considerations for ErbB-family TKI-associated diarrhea

**DOI:** 10.1007/s10549-018-05102-x

**Published:** 2019-01-22

**Authors:** Hope S. Rugo, Jack A. Di Palma, Debu Tripathy, Richard Bryce, Susan Moran, Elizabeth Olek, Linda Bosserman

**Affiliations:** 1 Department of Medicine (Hematology/Oncology), University of California San Francisco Helen Diller Family Comprehensive Cancer Center, 1600 Divisadero St., Box 1710, San Francisco, CA 94143-1710 USA; 2 Division of Gastroenterology, University of South Alabama College of Medicine, 75 S. University Blvd., Mobile, AL 36688 USA; 3 Department of Breast Medical Oncology, The University of Texas MD Anderson Cancer Center, 1515 Holcombe, Unit 1354, Houston, TX 77030 USA; 4 Puma Biotechnology, Inc., 10880 Wilshire Blvd. Suite 2150, Los Angeles, CA 90024 USA; 5 Puma Biotechnology, Inc., 701 Gateway Blvd, Suite 500, South San Francisco, CA 94080 USA; 6 City of Hope Medical Group, Inc, 1500 E Duarte Rd, Duarte, CA 91010 USA; 7 Present Address: QED Therapeutics, 421 Kipling St, Palo Alto, CA 94301 USA

**Keywords:** Cancer, Diarrhea, ErbB receptor, Tyrosine kinase inhibitor

## Abstract

**Purpose:**

Diarrhea is recognized as a common adverse event associated with tyrosine kinase inhibitors (TKIs), with those targeting the ErbB family of receptors being associated with the highest rate of diarrhea.

**Methods:**

This paper reviews data on the incidence, timing, and duration of diarrhea associated with US Food and Drug Administration-approved ErbB family-targeted TKIs from the published literature, and sets forth recommendations for management.

**Results:**

In the absence of anti-diarrheal prophylaxis the incidence of any-grade diarrhea varies and typically occurs early during the course of treatment. Although it is difficult to determine if the incidence and severity of diarrhea is related to inhibition of a particular kinase target because of the multi-targeted and overlapping activity of many agents, evidence suggests that second-generation TKIs with broader target profiles (i.e., afatinib, lapatinib, neratinib) result in a higher incidence of diarrhea compared with highly specific first- (erlotinib, gefitinib) or third- (osimertinib) generation agents. The mechanisms responsible for TKI-associated diarrhea are not fully understood and are likely multi-factorial, involving dysregulated ion transport, inflammation, and mucosal injury. Management strategies have been developed—and continue to be refined—to prevent and reduce the severity and duration of TKI-associated diarrhea. For agents associated with more significant symptoms, anti-diarrheal prophylaxis reduces the incidence and severity of diarrhea, and ongoing studies are evaluating specific strategies to further reduce incidence and duration of TKI-associated diarrhea.

**Conclusions:**

Continued investigations into risk factors and pharmacogenomic markers for diarrhea may further improve management of this common toxicity.

## Introduction

Currently, 37 different tyrosine kinase inhibitors (TKIs) are approved by the US Food and Drug Administration (FDA) for the treatment of various cancers (Table 1) [1, 2]. These orally administered targeted agents improve patient outcomes in a variety of settings; however, TKIs have a unique adverse event profile including gastrointestinal and cutaneous side effects that must be recognized and managed appropriately [3, 4].

**Table 1:** FDA-approved TKIs [1, 2]

Abemaciclib	Dabrafenib	Nilotinib
Acalabrutinib	Dasatinib	Osimertinib
Afatinib	Encorafenib	Palbociclib
Alectinib	Erlotinib	Pazopanib
Axitinib	Gefitinib	Ponatinib
Binimetinib	Ibrutinib	Regorafenib
Bosutinib	Idelalisib	Ribociclib
Brigatinib	Imatinib	Sorafenib
Cabozantinib	Lapatinib	Sunitinib
Ceritinib	Lenvatinib	Trametinib
Cobimetinib	Midostaurin	Vandetanib
Copanlisib	Neratinib	Vemurafenib
Crizotinib		

Diarrhea is a common adverse event associated with TKIs. The incidence of all-grade diarrhea in the absence of anti-diarrheal prophylaxis varies from 18 to 95% depending on the agent [5–12]. Diarrhea associated with TKIs can be severe and, if not properly managed, can lead to severe dehydration, dose reductions, and treatment interruptions or discontinuations. It is difficult to determine if the incidence and severity of diarrhea is related to inhibition of a particular kinase target because of the multi-targeted and overlapping activity of many agents [13]. TKIs associated with the highest rate of diarrhea are those targeting the ErbB family of receptors (ErbB1 [HER1, EGFR1], ErbB2 [HER2], ErbB3 [HER3], ErbB4 [HER4]), Bcr-Abl, phosphatidylinositol 3-kinase (PI3K) delta, and vascular endothelial growth factor. In this review, we will focus on the ErbB-family TKIs, some of which target multiple members of the ErbB family, but generally do not have target receptors beyond the ErbB family. Evidence suggests that second-generation agents, including afatinib, lapatinib, and neratinib, which have broader inhibitory profiles, result in a greater incidence of both all-grade and severe diarrhea compared with first-generation agents such as erlotinib and gefitinib that are highly specific to epidermal growth factor receptor (EGFR). For example, the incidence of all-grade diarrhea reported in phase 3 clinical trials of erlotinib, a selective EGFR TKI, was 18–55%, and grade ≥ 3 diarrhea was reported in 3–6% of patients [14, 15]. In contrast, the incidence of all-grade diarrhea associated with afatinib, an ErbB-family TKI that blocks signaling from EGFR, human epidermal growth factor receptor 2 (HER2), and human epidermal growth factor receptor 4 (HER4), was 75–95%, with grade ≥ 3 diarrhea in 10–14% of patients [5, 16, 17].

Signaling by the ErbB family of receptors plays a critical role in normal physiological functioning of cells; these receptors are expressed in a variety of epithelial, mesenchymal, and neuronal tissues and initiate a complex signaling cascade, which regulates proliferation, differentiation, migration, and apoptosis [18]. Overexpression, activating mutations, and autocrine stimulation of ErbB-family receptors—particularly EGFR and HER2—can lead to uncontrolled cell division via activation of multiple downstream signaling pathways [19–21], which is particularly common in breast and lung cancers. For example, up to 15% of Caucasian patients and 20–40% of Asian patients with non-small cell lung cancer (NSCLC) have an activating *EGFR* mutation [22, 23]. HER2-positive breast cancers represent approximately 20% of all breast cancers and tend to be more aggressive than HER2-negative breast cancers [24]. Inhibiting EGFR and HER2 with receptor-targeted TKIs is an important therapeutic approach in these cancers. Currently, there are 6 approved TKIs that primarily target the ErbB family; some agents inhibit multiple members of the ErbB receptor family with varying levels of target specificity and activity (Table 2).

**Table 2:** Inhibitory profiles of FDA-approved ErbB family-targeted TKIs approved for BC and NSCLC^a^ [25, 26]

Agent	Target(s)	IC_50_, nM
Gefitinib	EGFR	0.5–33
Erlotinib	EGFR	0.6–0.8
Lapatinib	EGFR	0.3–17
	HER2	6–25
Afatinib	EGFR	0.5
	HER2	14
	HER4	1
Neratinib	EGFR	92
	HER2	59
	HER4	NR
Osimertinib	WT EGFR	184
	T790M EGFR	1

*BC* breast cancer, *IC*_*50*_ drug concentration causing 50% inhibition of the desired activity in *in vitro* EGFR kinase assays, *EGFR* epidermal growth factor receptor, *HER2* human epidermal growth factor receptor 2, *HER4* human epidermal growth factor receptor 4, *NR* not reported, *NSCLC* non-small cell lung cancer, *TKI* tyrosine kinase inhibitors, *WT* wild type

^a^The measurement of IC_50_ values is dependent on the assay; direct, cross-assay comparisons should be interpreted carefully

This review focuses on the incidence and management of diarrhea associated with the following 6 FDA-approved TKIs that target the ErbB-family receptors EGFR and HER2 (as of 31 August 2018): gefitinib, erlotinib, lapatinib, afatinib, neratinib, and osimertinib. Future considerations for optimizing the management of TKI-associated diarrhea based on current knowledge about the pathophysiology of diarrhea will also be discussed.

## Literature search criteria and methods

Databases searched included Medline (last searched: March 6, 2018); American Society of Clinical Oncology abstracts (2011–2017); European Society for Medical Oncology abstracts (2012–2016); San Antonio Breast Cancer Symposium abstracts (2014–2017); International Association for the Study of Lung Cancer World Conference on Lung Cancer abstracts (2013–2016); clinicaltrials.gov; FDA oncology approvals. Search terms were “diarrhea” AND “target therapy” [substance name].

## Definition and differential diagnosis

The National Cancer Institute Common Toxicity Criteria for Adverse Events (NCI-CTCAE) provides standard grading for the severity of diarrhea, based primarily on the increase in the number of stools per day compared with baseline (Table 3) [27]. In clinical practice, these criteria, combined with patient assessment, laboratory data, and information obtained from symptom diaries about other physical symptoms such as fever, chills, and nausea, provide insight into the etiology and severity of the diarrhea, all of which are required for optimal patient management.

**Table 3:** Severity of diarrhea by grade according to the NCI CTC for Adverse Events (NCI-CTCAE v4) [27]

Grade	Description
1 (Mild)	Increase of < 4 stools per day over baseline; mild increase in ostomy output compared to baseline
2 (Moderate)	Increase of 4 to 6 stools per day over baseline; moderate increase in ostomy output compared to baseline
3 (Severe)	Increase of ≥ 7 stools per day over baseline; incontinence; hospitalization indicated; severe increase in ostomy output compared to baseline; limiting self-care ADL
4 (Life-threatening)	Life-threatening consequences; urgent intervention indicated
5 (Death)	Death

*ADL* activities of daily living, *AE* adverse event, *CTC* Common Toxicity Criteria, *NCI* National Cancer Institute

Based on the NCI-CTCAE grade and the presence or absence of additional symptoms, treatment-related diarrhea may be categorized as uncomplicated or complicated. Uncomplicated diarrhea is defined as grade 1 or 2 diarrhea without complicating signs or symptoms, including moderate to severe cramping, nausea, vomiting, decreased performance status, fever, sepsis, neutropenia, bleeding, and dehydration [28]. Mild to moderate (grade 1 or 2) diarrhea in the presence of at least 1 complicating factor, or diarrhea that is grade ≥ 3 is considered complicated [28].

When a patient experiences diarrhea during treatment, the first step is to rule out alternative causes [28]. The type of diarrhea is also important for proper management and control. Osmotic diarrhea is caused primarily by use of laxatives or inefficient digestion of certain food substances. In this case, stool output is proportional to the intake of the unabsorbable substrate and is usually not severe; normal stool output returns with discontinuation of causative agent or food. In secretory diarrhea, the type of diarrhea that commonly occurs in patients taking TKIs, the ion transport processes of the epithelial cells of the gastrointestinal tract are in a state of active secretion. Common causes of acute-onset secretory diarrhea (in a healthy individual not taking TKIs) are bacterial and viral infections of the gut. This should, of course, be ruled out in patients receiving targeted therapies, although infections are not common in this setting.

## Pathophysiology

Understanding the underlying pathologic cause of TKI-associated diarrhea is important for determining appropriate prophylactic and/or treatment regimens, as well as developing new therapeutic strategies to reduce incidence and severity. However, the exact mechanism(s) responsible for TKI-induced diarrhea still need to be fully elucidated, and may be multi-factorial in nature [6, 29].

EGFR and, to a much lesser extent, HER2 are expressed on healthy cells throughout the body including in the gastrointestinal tract [30]. EGFR is expressed at high levels in the basolateral membranes of epithelial cells and is critical for maintaining normal gut function through the regulation of ion transport, including the negative regulation of intestinal epithelial chloride secretion, which regulates the passive movement of water through the gastrointestinal lumen [31, 32]. Pre-clinical studies suggest that epidermal growth factor (EGF) decreases chloride secretion in T84 human colonic intestinal epithelial cells via a signaling cascade that involves protein kinase C and phosphatidylinositol 3-kinase [33]. Dysregulation of intestinal ion transport systems (e.g., through EGFR inhibition) can increase chloride secretion and lead to secretory diarrhea [32], suggesting a possible mechanism for EGFR TKI-associated secretory diarrhea. There is also pre-clinical evidence suggesting a role for HER2 in the inhibitory effect of EGF on epithelial chloride secretion through the formation of EGFR/HER2 heterodimers [34]. Inhibition of EGFR homodimer signaling vs EGFR/HER2 heterodimer signaling may explain the observed differences in the gastrointestinal effects of EGFR- and HER2-targeted agents; however, direct evidence of this effect in the gastrointestinal tract is lacking [35]. Nevertheless, the proposed mechanism involving the dysregulation of ion transport systems does provide researchers with a novel target for further development of management strategies for TKI-associated diarrhea (see discussion later in this article).

A number of other elegant pre-clinical models have been developed to further investigate the mechanism(s) responsible for TKI-associated diarrhea. An animal model of lapatinib-induced diarrhea that closely mimicked clinical symptoms did not support the hypothesis that disruption of chloride secretion contributes to lapatinib-induced diarrhea; lapatinib had no significant effect on serum chloride levels. In addition, there was no evidence of macroscopic or microscopic tissue injury within the jejunum or colon, suggesting that lapatinib alone does not cause epithelial damage. In contrast, combined treatment with paclitaxel and lapatinib in a rat model caused an increase in the incidence of severe diarrhea and weight loss compared with either agent alone, suggestive of intestinal tissue injury [36].

In contrast to the results reported for lapatinib [36], findings from models using other TKIs demonstrated TKI-induced direct mucosal damage apparently leading to diarrhea [37]. Mice that received a 10-day treatment course of gefitinib demonstrated significant atrophy of the small-intestinal wall, which resulted in a decreased absorptive surface area [38]. Similarly, intestinal mucosal damage was greater in mice treated with increasing doses of erlotinib over a 10-day period [39]. Finally, a rat model of neratinib-induced diarrhea suggested that inflammation may also contribute to the pathogenesis of diarrhea. In this model, neratinib increased histological damage in the distal ileum as well as production of pro-inflammatory cytokines, such as interferon-ɣ, in the ileum [40].

Treatment with budesonide, a corticosteroid with low oral bioavailability, decreased the number of days that the rats had diarrhea and reduced histopathological injury induced by neratinib [40]. In addition, budesonide reduced the activity of the inflammatory marker, myeloperoxidase, and increased levels of interleukin-4, an anti-inflammatory cytokine, while decreasing levels of interferon-ɣ. The effects of budesonide and colesevelam, a bile acid sequestrant, are currently being investigated in an ongoing Australian rat model study of neratinib-induced diarrhea.

Multiple other mechanisms—or a combination of mechanisms—have also been proposed as potential causes of TKI-induced diarrhea, including changes in gut motility, damage in the colonic crypts, and altered interstitial microflora [6]. Unfortunately, inter-species differences between model systems (and individuals), in conjunction with variability in the target profile of individual TKIs, make it difficult to pinpoint the precise mechanism(s) responsible for the associated diarrhea.

## Clinical experience

Diarrhea, a common side effect of many cancer treatments, including chemotherapeutic agents, targeted therapies, and pelvic radiotherapy, is one of the most common adverse events reported during treatment with a TKI, and can be dose-limiting for TKIs that block EGFR signaling [41]. Effective clinical management of TKI-associated diarrhea requires a clear understanding of incidence, severity, onset, and duration. The incidence of all-grade diarrhea reported for gefitinib, erlotinib, afatinib, and osimertinib in NSCLC and lapatinib and neratinib in breast cancer varies from 18 to 95% depending on the agent (Table 4) [5–12]. Among TKIs targeting the ErbB family of receptors, it appears that the incidence and severity of diarrhea increase with the increasing breadth of targets. Newer, second-generation TKIs were developed with broader target profiles in an attempt to improve the duration of response and overcome resistance to acquired mutations. However, the benefits of these agents come at a cost, with the potential for a higher incidence of adverse events compared with more selective reversible TKIs. Diarrhea was reported in 87–95% of patients receiving afatinib and neratinib, both of which are second-generation irreversible TKIs [16, 29, 42], compared with 18–69% of patients receiving erlotinib and gefitinib [9, 12]. The severity of diarrhea also varies with target profile, with grade ≥ 3 diarrhea reported in up to 25% of patients treated with more focally targeted first-generation TKIs and up to 40% of patients treated with broadly targeted second-generation TKIs.

**Table 4:** FDA-approved oncology ErbB family-targeted TKIs approved for BC and NSCLC by frequency of associated diarrhea

Agent	Year approved	Cancer	Diarrhea, %^a^		Median time, days	
			All-grade	Grade 3/4	Onset	Duration
Afatinib [5, 6]	2013	NSCLC	87–95	5–17	7–14^b^	NR
Neratinib [7, 8]	2017	HER2 + BC	95	40^c^	2	5^d^
Lapatinib [9]	2007	BC	65	14	~ 6^e^	4–5
Osimertinib [11]	2015	NSCLC	41	1	NR	NR
Gefitinib [12]	2003	NSCLC	27–69	1–25	NR	NR
Erlotinib [10]	2004	NSCLC; pancreatic cancer	18–68	1–12	12–32	NR

*BC* breast cancer, *EGFR* epidermal growth factor receptor, *HER2* + human epidermal growth factor receptor 2-positive, *NR* not reported, *NSCLC* non-small cell lung cancer, *TKI* tyrosine kinase inhibitors

^a^From phase 3 clinical trials

^b^In 50–62% of patients and 71% of patients, respectively, across clinical trials

^c^Without loperamide prophylaxis; 1 patient experienced grade 4 diarrhea

^d^For grade 3 diarrhea. Duration represents cumulative duration over the course of a year

^e^In almost half of the patients with diarrhea

TKI-associated diarrhea typically occurs early during a course of treatment. For example, in studies without anti-diarrheal prophylaxis, diarrhea associated with afatinib typically occurs in 50–62% of patients within the first 7 days of therapy and in 71% of patients by 14 days after initiating treatment [6]. Similarly, in the phase 3 ExteNET trial (which did not include anti-diarrheal prophylaxis), 40% of patients taking neratinib experienced grade 3 diarrhea after a median of 8 days (interquartile range 4–33), lasting a median of 5 days (interquartile range 2–9) per patient, with a median of two grade ≥ 3 diarrhea events per patient over the course of 1 year of treatment [42].

In clinical trials, TKI-associated diarrhea is a leading cause of dose reductions and treatment discontinuations. In the pivotal trial evaluating afatinib for metastatic NSCLC, diarrhea resulted in dose reductions and treatment discontinuations in 20% and 1.3% of patients in the afatinib arm, respectively [16]. Similarly, in the ExteNET trial, 26% of patients taking neratinib for 1 year without anti-diarrheal prophylaxis required dose reductions due to diarrhea and 17% discontinued treatment. Both dose reductions and discontinuations occurred early (median of 20 days after treatment initiation, interquartile range 9–56 days) [42]. Importantly, diarrhea associated with neratinib resolved quickly when treatment was held and was rarely associated with serious consequences (e.g., renal insufficiency) [43].

The safety profile is different for TKIs that specifically inhibit mutant targets and have a low affinity for wild-type receptors (e.g., osimertinib, a third-generation TKI that potently inhibits T790M EGFR but has a low affinity for wild-type EGFR). These agents are effective in patients whose tumors have acquired resistance to earlier-generation, more broadly specific TKIs and may reduce the adverse effects of inhibiting wild-type receptors and/or off-target effects in normal tissue. The reported incidence of all-grade and grade ≥ 3 diarrhea in patients treated with osimertinib was 41% and 1%, respectively, in the pivotal trial in patients with NSCLC [44]. Osimertinib-associated diarrhea may be due to low-level inhibition of wild-type EGFR [45] and/or non-EGFR related mechanisms that contribute to diarrhea, including direct mucosal damage, resulting in decreased absorption of water [37]. This hypothesis is consistent with the increased incidence of diarrhea observed with escalating doses of osimertinib in the phase 1 clinical trial [46]. Full investigations into third-generation agents are underway; however, toxicity is likely to vary by agent and target.

In summary, although diarrhea is a common side effect of all TKIs, data suggest that second-generation inhibitors are associated with the highest incidence of diarrhea, likely due to broader and off-target effects compared with first-generation agents.

## Management strategies

Numerous consensus guidelines and clinical recommendations have been published on the management of cancer treatment-induced diarrhea [47–50] and, recently, agent-specific TKI-induced diarrhea [6, 29, 51, 52]. Diarrhea management recommendations are also included in the US prescribing information for these agents (Table 5) [5, 7, 9–12]. Cancer treatment-induced diarrhea management recommendations and those for TKI-associated diarrhea are similar, with management based on severity and associated complications [48]. For example, most cases of uncomplicated grade 1 or 2 diarrhea can be managed effectively with self-administered anti-diarrheal agents such as loperamide, diphenoxylate/atropine, or racecadotril. Loperamide acts as an agonist on opioid receptors in the gastrointestinal tract, decreasing gut motility and inhibiting ion transport, but is minimally absorbed and thus has limited central nervous system effects [49, 53]. Racecadotril is an enkephalinase inhibitor with proven efficacy in models of hypersecretory diarrhea, but it does not enter the brain after oral administration and therefore has no central nervous system effects [54, 55]. In contrast, patients with grade ≥ 3 and/or medically complicated diarrhea require more aggressive management, including treatment interruptions and re-initiation of treatment at a reduced dose, in addition to anti-diarrheal therapies. Medically complicated diarrhea may include moderate to severe cramping, grade ≥ 2 nausea/vomiting, decreased performance status, fever, sepsis, neutropenia, frank bleeding, and dehydration [48], each of which needs to be evaluated and treated. The early identification and proactive management of TKI-associated diarrhea may minimize the occurrence of high-grade events and further complications that lead to treatment discontinuations or hospitalizations.

**Table 5:** Diarrhea management recommendations available in the US prescribing information for ErbB family-targeted TKIs approved for BC and NSCLC

Agent	Anti-diarrheal treatment (e.g., loperamide)	Dose reductions	Dose interruptions^a^	Anti-diarrheal prophylaxis	Patient education/information	Supportive care
Gefitinib [12]			✓			
Erlotinib [10]	✓	✓	✓		✓	
Lapatinib [9]	✓	✓	✓		✓	✓
Afatinib [5]	✓	✓	✓		✓	
Neratinib [7]	✓	✓	✓	✓	✓	✓
Osimertinib [11]					✓	

Detailed recommendations can be found in the prescribing information for each agent

*BC* breast cancer, *NSCLC* non-small cell lung cancer, *TKI* tyrosine kinase inhibitors

^a^Management strategies include withholding treatment for severe (grade 3/4) or persistent (lasting up to 14 days) diarrhea, and resuming treatment (potentially at a lower starting dose) when diarrhea is grade ≤1

### Anti-diarrheal prophylaxis

Although anti-diarrheal prophylaxis is not recommended for all TKIs before treatment initiation, prophylaxis has demonstrated success at reducing the incidence, severity, and duration of diarrhea for agents with high upfront risk and continues to be investigated. The phase 3 ExteNET trial, evaluating 1 year of neratinib as extended adjuvant therapy in HER2 + breast cancer, did not mandate the use of anti-diarrheal prophylaxis, instead recommending treatment with anti-diarrheal agents and/or dose modifications after symptom onset. Even though most occurrences of diarrhea were self-limiting (resolving quickly after drug hold), grade 3 diarrhea occurred in 40% of patients and 17% of patients discontinued neratinib due to gastrointestinal adverse events. In patients with metastatic breast cancer or NSCLC, loperamide administered during the first month of neratinib treatment (with the first dose and continued through the first cycle of treatment) effectively reduced the rates of grade 3 diarrhea (0–17% across studies) [56–58]. As a result, anti-diarrheal prophylaxis with loperamide from the first dose through two cycles of treatment, along with patient education, is the recommended approach for patients receiving neratinib as extended adjuvant therapy [7]. The effectiveness of this approach, as well as other anti-diarrheal agents, continues to be investigated in this setting.

An international, open-label, sequential-cohort, phase 2 study (CONTROL) in HER2 + breast cancer patients receiving extended adjuvant neratinib therapy [59–61] is currently investigating the effects of several prophylactic strategies in reducing neratinib-associated diarrhea. The study cohorts include the following: mandatory loperamide prophylaxis (actual n = 137); loperamide + budesonide (actual n = 64); loperamide + the bile acid sequestrant colestipol (actual n = 136); colestipol + prn loperamide (actual n = 104); or a dose-escalation strategy (target n = 64, just starting to enroll) on neratinib-associated diarrhea [60, 61] (Table 6) [59, 62, 63].

**Table 6:** Ongoing clinical trials of EGFR/HER2-targeted TKI-associated diarrhea

Agent	Phase	Estimated enrollment	Design	Title
Lapatinib [62]	2	140	Randomized, parallel, open label	A randomized, multicenter, open-label, phase 2 study of prophylactic octreotide to prevent or reduce the frequency and severity of diarrhea in subjects receiving lapatinib with capecitabine for the treatment of mBC (NCT02294786)
Neratinib [63]	2	23	Single-arm, open label	An open-label study to characterize the incidence and severity of diarrhea in patients with early-stage HER2 + BC treated with adjuvant trastuzumab and neratinib followed by neratinib monotherapy, and intensive anti-diarrheal prophylaxis (NCT03094052)
Neratinib [59]	2	240	Non-randomized, sequential cohort, open label	An open-label study to characterize the incidence and severity of diarrhea in patients with early-stage HER2 + BC treated with neratinib and intensive loperamide prophylaxis (CONTROL) (NCT02400476)

*BC* breast cancer, *EGFR* epidermal growth factor receptor, *HER2* + human epidermal growth factor receptor 2-positive, *mBC* metastatic breast cancer, *TKI* tyrosine kinase inhibitors

Interim data demonstrated that loperamide prophylaxis modestly reduced the incidence of grade ≥ 3 neratinib-associated diarrhea compared with that reported in the phase 3 ExteNET trial without anti-diarrheal prophylaxis (30.7% vs 39.9%, respectively). Adding budesonide or colestipol further reduced the incidence of grade ≥ 3 diarrhea (26.6% and 10.8%, respectively) [60, 61], suggesting that the etiology of neratinib-associated diarrhea may involve inflammation and/or bile acid malabsorption. Compared with the ExteNET trial, prophylaxis with loperamide alone also reduced the median cumulative duration of diarrhea grade ≥ 2 (4 vs 10 days) and grade ≥ 3 (3 vs 5 days) and reduced the median number of diarrhea episodes (any grade) per patient (2 vs 8 episodes). Of note, no grade 4 diarrhea events have occurred. These data suggest that effective anti-diarrheal prophylaxis may help improve the tolerability of neratinib, and ongoing cohorts are exploring colestipol alone or a neratinib dose-escalation strategy.

Investigations are underway to develop pharmacologic management approaches targeting chloride channels, which may be an important mechanism associated with EGFR inhibitors. Crofelemer, an established anti-diarrheal agent for other conditions, inhibits chloride channels in the membrane of epithelial cells [64] and has been investigated in patients with breast cancer receiving targeted therapies in combination with chemotherapy [65, 66]. An ongoing study is investigating anti-diarrheal prophylaxis with crofelemer in HER2-positive patients treated with adjuvant trastuzumab and neratinib followed by neratinib monotherapy (Table 5) [63].

Based on the incidence and severity of lapatinib-associated diarrhea, studies continue to assess the clinical utility of anti-diarrheal prophylaxis. A small retrospective analysis of 44 patients treated with lapatinib suggested that anti-diarrheal prophylaxis may be an effective strategy for patients to achieve therapy goals without interruptions or dose adjustments [67]. An ongoing, randomized, phase 2 study with an estimated enrollment of 140 patients will assess the effect of prophylactic octreotide, a somatostatin analog and established anti-diarrheal agent for cancer treatment-induced diarrhea, to prevent or reduce the frequency and severity of diarrhea in subjects receiving lapatinib in combination with capecitabine for the treatment of HER2 + metastatic breast cancer (Table 5) [62].

Further research is needed to reduce constipation associated with prophylactic regimens and optimize tolerability. For example, in the CONTROL trial, rates of grade 1/2 constipation were 42.3%/14.6% in patients receiving loperamide prophylaxis alone, 62.5%/12.5% with loperamide plus budesonide, and 53.3%/9.2% with loperamide plus colestipol [61]. An ongoing area of investigation in the CONTROL trial is whether anti-diarrheal prophylaxis will have a significant impact on health-related quality of life (HRQoL) [59].

### Monitoring

Clear communication between patients and physicians is critical to ensure accurate reporting and characterization of symptoms, exclude other potential causes, and to initiate therapy adjustments as needed. Close patient monitoring is critical for management of diarrhea during the first few weeks of therapy because diarrhea generally occurs within the first week for most TKIs (Table 3) [27]. Patients should be educated that diarrhea is a common side effect of TKIs and about the potential consequences of diarrhea, including dehydration, electrolyte imbalances, and renal insufficiency. Patients should be instructed on the use of anti-diarrheal treatment regimens either as prophylaxis or in case of mild to moderate diarrhea per treatment guidelines, and to monitor the frequency of bowel movements. In addition, patients should be advised to inform their healthcare provider immediately if diarrhea develops (i.e., more than 2–3 episodes per day), for severe or persistent diarrhea, or if diarrhea is associated with weakness, dizziness, or fever. In clinical practice, patient diaries combined with proactive triage management and a thorough evaluation of treatment-emergent diarrhea help to ensure that appropriate management measures are taken.

### Intervention

Proactive management of treatment-related diarrhea is recommended to reduce the incidence, duration, and severity. Loperamide is the mainstay for the pharmaceutical management of uncomplicated mild to moderate diarrhea and is recommended in the prescribing information after the onset of diarrhea or as upfront prophylaxis [68]. In some cases, diarrhea persists despite administration of anti-diarrheal agents given prophylactically or at first onset of diarrhea. In this case, TKI treatment should be withheld until diarrhea has resolved to baseline or grade ≤1 and may be reinitiated per labeling. In cases of severe or persistent diarrhea, supportive care including the administration of oral or intravenous electrolytes and fluids, addition of alternative anti-diarrheal agents (e.g., diphenoxylate–atropine, octreotide, or tincture of opium), and diet modification are recommended [48]. Evaluation for infectious diarrhea or other causes should also be considered along with appropriate use of anti-infectives.

In all cases, patient education is critical to help patients identify the signs and symptoms of diarrhea and stress the need for urgent action (i.e., loperamide administration and contacting the patient’s physician for severe or persistent diarrhea).

## Future directions

Novel pharmacologic approaches to TKI-associated diarrhea based on its pathophysiology will improve the quality of care and further optimize diarrhea management approaches for patients receiving these targeted therapies [59–61]. Research is needed to better understand risk factors for TKI-associated diarrhea, with the goal of identifying those patients at greater risk and to manage diarrhea both proactively and emergently. A proof-of-principle study designed to quantify the risk factors for diarrhea in breast cancer patients treated with lapatinib identified risk factors including advanced age, starting treatment in the spring, a higher incidence of skin metastases, and grade 1 diarrhea in earlier treatment cycles [69]. Pharmacogenomic studies may help to further elucidate the pathophysiology of TKI-induced diarrhea and allow for the identification of vulnerable patients.

## Abbreviations

EGF: Epidermal growth factor
EGFR: Epidermal growth factor receptor
FDA: US Food and Drug Administration
HER: Human epidermal growth factor receptor
NSCLC: Non-small cell lung cancer
TKI: Tyrosine kinase inhibitors
